# Enhanced laser-induced PEDOT-based hydrogels for highly conductive bioelectronics

**DOI:** 10.1093/nsr/nwaf136

**Published:** 2025-04-04

**Authors:** Hao Zhou, Ziguan Jin, Yuhong Xu, Yuyao Lu, Zhuoheng Xia, Fan Yang, Qianglong Wu, Yang Gao, Jun Yin, Jianhua Zhang, Chujun Ni, Bin Zhang, Yong He, Huayong Yang, Kaichen Xu

**Affiliations:** State Key Laboratory of Fluid Power & Mechatronic Systems, School of Mechanical Engineering, Zhejiang University, Hangzhou 310023, China; State Key Laboratory of Fluid Power & Mechatronic Systems, School of Mechanical Engineering, Zhejiang University, Hangzhou 310023, China; State Key Laboratory of Fluid Power & Mechatronic Systems, School of Mechanical Engineering, Zhejiang University, Hangzhou 310023, China; State Key Laboratory of Fluid Power & Mechatronic Systems, School of Mechanical Engineering, Zhejiang University, Hangzhou 310023, China; Center for Plastic & Reconstructive Surgery, Department of Stomatology, Zhejiang Provincial People's Hospital, Affiliated People's Hospital, Hangzhou Medical College, Hangzhou 310023, China; Center for Plastic & Reconstructive Surgery, Department of Stomatology, Zhejiang Provincial People's Hospital, Affiliated People's Hospital, Hangzhou Medical College, Hangzhou 310023, China; Center for X-Mechanics, Department of Engineering Mechanics, Zhejiang University, Hangzhou 310027, China; Center for X-Mechanics, Department of Engineering Mechanics, Zhejiang University, Hangzhou 310027, China; State Key Laboratory of Fluid Power & Mechatronic Systems, School of Mechanical Engineering, Zhejiang University, Hangzhou 310023, China; State Key Laboratory of Fluid Power & Mechatronic Systems, School of Mechanical Engineering, Zhejiang University, Hangzhou 310023, China; Eye Center, Affiliated Second Hospital, School of Medicine, Zhejiang University, Hangzhou 310009, China; State Key Laboratory of Fluid Power & Mechatronic Systems, School of Mechanical Engineering, Zhejiang University, Hangzhou 310023, China; State Key Laboratory of Fluid Power & Mechatronic Systems, School of Mechanical Engineering, Zhejiang University, Hangzhou 310023, China; State Key Laboratory of Fluid Power & Mechatronic Systems, School of Mechanical Engineering, Zhejiang University, Hangzhou 310023, China; State Key Laboratory of Fluid Power & Mechatronic Systems, School of Mechanical Engineering, Zhejiang University, Hangzhou 310023, China

**Keywords:** conductive hydrogels, PEDOT:PSS, laser-induced phase separation, metastable liquid–liquid contact, implantable bioelectronics

## Abstract

Conductive hydrogels—particularly poly(3,4-ethylenedioxythiophene) (PEDOT)-based hydrogels—possess mechanical properties comparable to biological tissues and superior biocompatibility. Laser treatment affords a promising approach for the development of well-patterned PEDOT bioelectrodes. However, the weak photothermal conversion of pristine PEDOT-based solution results in very limited phase separation and thus low conductivity. Here, we report an enhanced laser-induced PEDOT (ELIP)-based hydrogel via a metastable liquid–liquid contact (MLLC) strategy. Such MLLC pretreatment renders the extension of PEDOT chains with an increase in the conjugation length, which greatly improves the light absorbance and photothermal conversion capability, achieving a conductivity of ≤955 S/cm and ∼3 μm-precision patterning. The laser treatment with an intensive and instantaneous thermal effect also elevates the interfacial adhesion and electrochemical stability of the proposed ELIP in physiological environments. Serving as the stimulator and signal recording for bioelectronic devices, the patterned ELIP showcases potential in nerve-conduction blocks for pain treatments.

## Teaser

This study presents an enhanced laser-induced PEDOT hydrogel with improved conductivity and precision via metastable liquid-liquid contact processing, offering promising bioelectronic applications in nerve conduction blocking.

## INTRODUCTION

Bioelectronic interfaces with desired physical and chemical properties play vital roles in providing multimodal physiological information and medical therapies [1–6]. Among the diverse soft electronic materials that are used to construct such bioelectronic devices, conductive hydrogels present mechanical properties that are similar to those of biological tissues [7–10]. In particular, poly(3,4-ethylenedioxythiophene):poly(styrene sulfonate) (PEDOT:PSS)-based hydrogels—known for their superior biocompatibility as well as electrical and/or ionic conductivity—serve as promising bioelectrode alternatives [11–13]. Various strategies are performed to enhance their conductivity and electrochemical stability [14–17]. Thermal annealing can maintain the conductivity, but the resulting films are inadequate for ensuring stability in liquid environments due to incomplete phase separation [18,19]. To address this limitation, chemical doping is often employed to enhance the carrier mobility via PEDOT crystallization [20–25]. Nevertheless, it is constrained by the dispersion of dopants, and residual dopants may compromise biocompatibility and long-term stability. A cross-linking strategy requires additional acid immersion to achieve further conductivity improvement [26–30]. Besides the conductivity and electrochemical stability, a fine spatial resolution is also vital for the development of implantable bioelectronic interfaces.

Laser-induced phase separation of PEDOT:PSS-based hydrogels enables the desired conductivity, stable mechanical properties and precise patterning, offering a promising solution to current challenges by virtue of the multitasking processing capability of laser means [31,32]. Specifically, laser irradiation disrupts the electrostatic equilibrium between PEDOT^+^ and PSS^−^, while promoting physical connections between the PEDOT domains [33]. This gives rise to phase separation, which is highly crucial for enhancing conductivity of the hydrogel by creating more interconnected PEDOT pathways. Nevertheless, the original PEDOT:PSS aqueous solution suffers from limited phase separation that is caused by its low photothermal conversion capability. Current approaches mainly involve the incorporation of noble metals based on the plasmonic effect [34] or post-treatment with organic solvents [35] for better organization of the PEDOT domains. However, the electrical conductivity of the resulting PEDOT:PSS films is still limited.

Here, we report an enhanced laser-induced PEDOT (ELIP)-based conductive hydrogel for implantable bioelectronics. We overcome the weak photothermal conversion of the original PEDOT:PSS solution via metastable liquid–liquid contact (MLLC) processing. The pretreated PEDOT:PSS solution with an increase in the conjugation length exhibits enhanced absorbance in the visible-light region. This enhanced absorbance apparently boosts the photothermal conversion property of the film that is irradiated by a continuous-wave 532-nm laser, resulting in a high conductivity of ≤955 S/cm. A resolution of 3 μm in line width is realized with enhanced interfacial adhesion and electrochemical stability. As a demonstration, the patterned ELIP electrodes were employed to validate their applications in nerve-conduction block, showcasing their capability for signal stimulating and recording. With the desired conductivity and biocompatibility, the proposed ELIP hydrogels hold promise for applications of wearable and implantable bioelectronics.

## RESULTS AND DISCUSSION

### Design and fabrication of highly conductive laser-induced hydrogels

**Figure 1.**
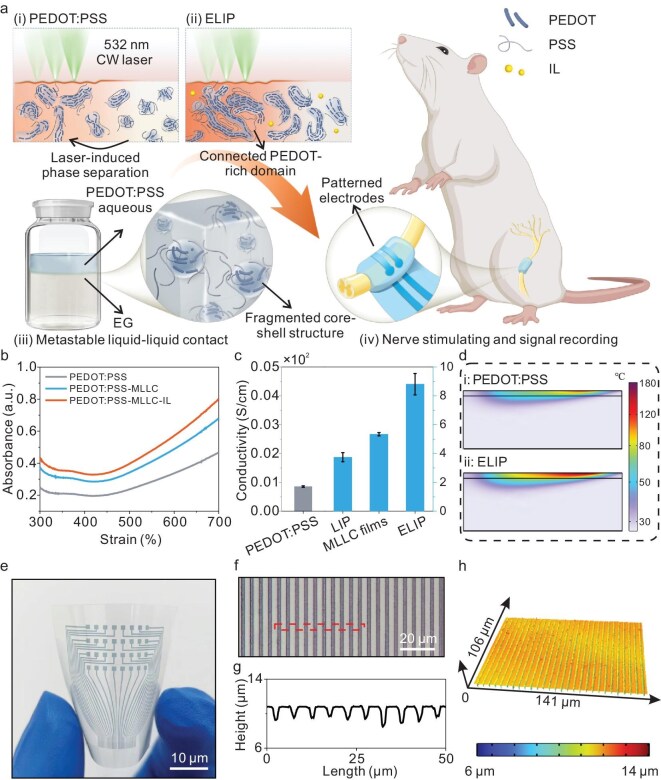
Design and fabrication of highly conductive hydrogels. (a) Schematic of design principles of bioelectrodes via MLLC coupled with laser-induced phase separation for implantable nerve stimulation and signal recording. (b) Ultraviolet-visible absorption spectra of pure PEDOT:PSS, MLLC ink and MLLC ink optimized with ionic liquid (IL). (c) Electrical conductivity of PEDOT:PSS and MLLC films with and without laser processing (*n* = 5). (d) Calculated thermal distribution inside the PEDOT:PSS and MLLC films. (e) Photo of enhanced laser-induced PEDOT:PSS electrodes array patterned on a PET substrate. (f) Optical image, (g) corresponding height profile and (h) 3D topography of patterned ELIP bioelectrodes array with a width of ∼3 μm on the PET substrate.

To achieve biocompatible conductive backbones for soft bioelectronics, a strategy of ELIP-based hydrogels is proposed (Fig. 1a). Typically, limited phase separation tends to take place for pure PEDOT:PSS films due to the relatively small photothermal absorption (Fig. 1a(i)). However, the MLLC films are endowed with more extended and connected PEDOT domains with a higher photothermal conversion (Fig. 1a(ii)). The MLLC process is driven by the density difference that leads to a delamination phenomenon after the aqueous PEDOT:PSS is dripped into the ethylene glycol (EG) solution (Fig. 1a(iii) and Fig. S1). The lower EG gradually diffuses upward to form a water–EG solvent. The polarity of the EG can disrupt the initial core–shell structure of the PEDOT:PSS. Meanwhile, the excess PSS that is diffused into the lower EG solution and the upper part of the ink is extracted due to the preliminary phase separation, producing a larger proportion of PEDOT content [36]. As a result of this reorganization, the PEDOT chains became more aligned and interconnected. This improved molecular ordering enhances the π–π stacking interactions, leading to an increase in the conjugation length. As the PEDOT absorption band ranges from ∼300 to 700 nm, whereas that of PSS is <300 nm, such MLLC-processed PEDOT:PSS ink exhibited a higher absorbance within the visible-light spectrum (Fig. 1b).

Generally, the absorbance characteristic (*A*) is highly associated with the ability of materials to absorb laser energy. The spatial heat transfer under laser interaction can be described by using the following equation (Note S2):


(1) \begin{eqnarray*}
&&Q\left( {x,y,z,t} \right) = A \cdot {I_0}{\left( {\frac{{{\omega _0}}}{{\omega \left( z \right)}}} \right)^2} \\
&& \times\, \exp \left\{ { - \frac{{{{ [x - {\mu_x\!}\left(t \right)] }^2} + {{[y - {\mu _y\!}\left( t \right)]}^2}}}{{\omega {{\left( z \right)}^2}}}} \right\} \cdot t, \\
\end{eqnarray*}

where $Q$ is the heat within the film at position (*x*, *y*, *z*) at time *t*, ${I_0}$ is the maximum intensity at the center of the beam, $\omega $ is the radius of the light spot and (*μ_x_*(*t*), *μ_y_*(*t*)) is the center coordinates at time *t*. Considering this equation, the heat distribution inside the PEDOT:PSS films is directly influenced by the absorbance *A*, as well as other thermal parameters. Therefore, this higher absorbance allows the MLLC films to achieve more effective photothermal conversion under the same laser conditions, facilitating enhanced phase separation within the material. Besides, a tiny quantity of ionic liquid was added to thicken the ink to prepare the uniform films, in addition to further increasing the absorbance (Fig. S2). With optimized continuous-wave (CW) visible laser parameters, the conductivity of the ELIP hydrogels could reach 955 S/cm, which was 2.36 times higher than that of the laser-induced PEDOT:PSS (LIP) without the precursor pretreatment (Fig. 1c). After swelling in water for 1 h, the conductivity decreased to 223.4 S/cm under hydrated conditions (Fig. S3).

To numerically perform the heat-transfer analysis of laser irradiation on the pristine PEDOT:PSS and MLLC films, the thermal conductivity and heat capacity were first characterized (Figs S4 and S5). The thermal conductivity of the MLLC films, which were obtained by using the hot-disk method, was slightly higher than that of the pristine PEDOT:PSS. Additionally, the MLLC films exhibited a higher phase-transition temperature based on heat-capacity data. This indicates that the MLLC films can withstand a higher temperature to achieve further phase separation. Subsequently, thermal distribution inside the films was calculated based on the finite element method (Fig. 1d). The simulations revealed that the higher photothermal absorbance and conversion efficiency of the MLLC films created a more intense thermodynamic environment. This is a crucial factor that contributes to the enhanced electrical conductivity. The ability of MLLC films to generate and maintain higher internal temperatures supports more efficient laser-induced phase separations, rendering them highly suitable for bioelectronic applications that require robust conductivity and stability.

Furthermore, the ELIP-based hydrogels can be patterned to micro-sized designs by using a femtosecond (fs) laser system to realize the maskless fabrication of high-precision electrodes. As demonstrated in Fig. 1e, a flexible and almost transparent circuit is achieved on a polyethylene terephthalate (PET) film, with uniformly arranged microelectrodes. In addition, the films were subtractively patterned down to nearly 3 μm with a spacing of ∼2 μm, and periodic and stable stripes were maintained on the surface (Fig. 1f and g). The high precision of this fs laser system ensures that, even at such a small scale, the patterns remain consistent and well defined, as displayed in the 3D surface topography (Fig. 1h).

### Mechanisms and characterizations of ELIP bioelectrodes

**Figure 2.**
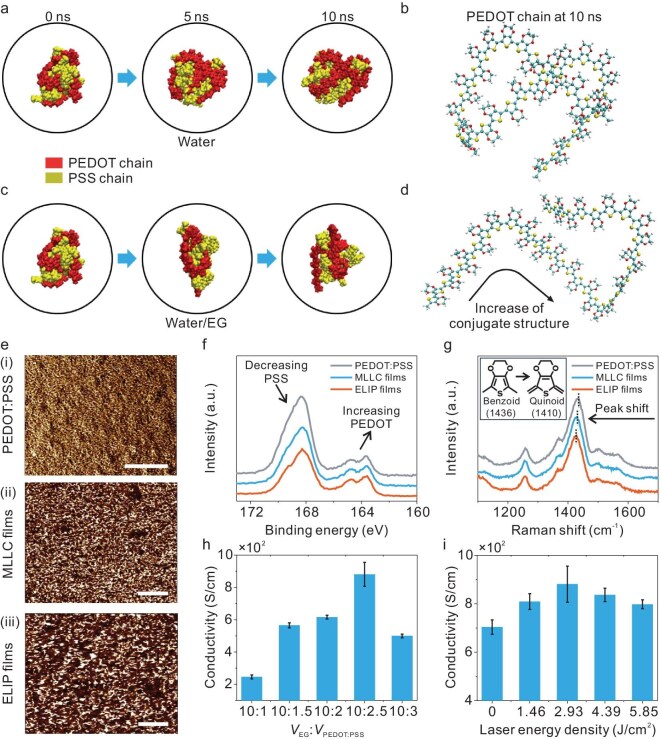
Molecular morphology simulations and characterization of ELIP bioelectrodes. (a) MD models and (b) morphology of PEDOT chains in water. (c) MD models and (d) morphology of PEDOT chains in water–EG solution. (e) AFM phase images of pure PEDOT:PSS, MLLC films and ELIP hydrogels. Scale bars, 1 μm. (f) XPS spectra and (g) Raman spectra of pure PEDOT:PSS, MLLC films and ELIP hydrogels. (h and i) Electrical conductivity of ELIP hydrogels as functions of volume ratios of EG to PEDOT:PSS (h) and laser energy density (i) (*n* = 5).

To substantiate the potential mechanisms by which such ELIP hydrogels perform better electrically than the original PEDOT:PSS films, simulations and characterizations were conducted. First, simulations of molecular dynamics were employed to calculate the transition of PEDOT and PSS chains in the water and water–EG systems, respectively (Fig. 2a and c). In terms of the water system, 10-ns calculations present almost no change in the distribution of the two phases, showing mutual entanglement and aggregation between the two components. This indicates no discernible phase separation. In contrast, during the simulation of the MLLC process that was initiated by the water–EG, the condensed phase gradually unfolded. The PEDOT chains progressively separated from the condensed phase, resulting in a tendency for phase separation (Fig. 2c). It is worth noting that the morphology of the PEDOT chains changed from a coiled state (Fig. 2b) to an extended state (Fig. 2d), allowing the conjugation length to increase in the PEDOT domain and π–π stacking regions. The expansion of the PEDOT domains and stackings enabled the electronic transitions and light adsorption in the visible range to improve [37,38]. Thus, the PEDOT:PSS films after the MLLC process further enhanced photothermal conversion and thermal absorption upon laser irradiation.

To further investigate the phase variation of the PEDOT:PSS-based films at each state, characterizations by using an atomic force microscope (AFM), X-ray photoelectron spectroscope (XPS) and Raman spectroscope were performed. The degree of phase separation was apparently observed in the phase images of the AFM, in which the bright regions represented PEDOT-rich domains and the dark regions referred to PSS-rich domains (Fig. 2e and Fig. S6). The untreated PEDOT:PSS films exhibited PEDOT-rich regions primarily in a dispersed and granular form, with few interconnections between these bright domains. This resulted in a lack of continuous conductive pathways and much lower conductivity. After the process of MLLC, the PEDOT-rich areas became more noticeable and began to connect into localized areas, bridging the partially isolated PEDOT grains. This formation can effectively enhance laser-matter interactions compared with untreated thin films, as in the aforementioned discussions. As displayed in Fig. 2e (iii), the MLLC film after laser treatment (i.e. ELIP) presented a further evolved morphology with the formation of a highly interconnected PEDOT network. Such a robust network-like distribution in the PEDOT enables improvement in the charge transport and significantly boosts the overall conductivity.

Subsequently, the ratio of PEDOT to PSS was investigated by using XPS (Fig. 2f). Typically, the S(2p) peaks of the PSS chain fall between 171 and 167 eV, whereas those of the PEDOT chain range from 167 to 163 eV [39,40]. The intensity of the PSS-related peak at ∼168 eV decreased gradually from the untreated PEDOT:PSS to the ELIP hydrogels, indicating a reduction in the PSS content. In contrast, the intensity of the PEDOT-related peak at ∼164 eV increased after both the MLLC process and the laser scanning, highlighting an enhanced PEDOT ratio. This variation trend suggests an effective modulation of the PEDOT ratio, improving the conductive networks in the treated films.

Another method by which to examine the molecular state of the PEDOT domains is Raman spectroscopy (Fig. 2g and Fig. S7). In general, a quinoid structure (1410 cm^−1^) with relatively high electrical conductivity and a benzoid structure (1436 cm^−1^) with relatively low electrical conductivity make up the chemical structures of the PEDOT crystallites. As can be seen, there is an obvious peak shift to the left in the Raman spectra of treated samples. By fitting and integrating these peaks, the ratio of the quinoid to benzoid structure gradually changes from 0.81 to 1.19 (Fig. S7). This indicates that the PEDOT morphology switches to linear structures from coiled structures after laser treatment. Such structural shifts match well with the calculations of the aforementioned molecular dynamics simulations, further confirming the morphological transformation. In sum, the thermal effect of the CW laser tends to stretch the molecular structure and extend the connection domains of the PEDOT, which can significantly enhance the conductivity of the ELIP.

Furthermore, the conductivity of the enhanced laser-treated MLLC films can be optimized by adjusting the volume ratio between the PEDOT:PSS and the EG (Fig. 2h). The conductivity reaches its maximum when the volume ratio of EG to PEDOT:PSS is 4:1 (Fig. 2h and Fig. S8). This suggests that an appropriate EG volume facilitates optimal phase separation and the formation of a highly conductive network. Excessive EG dispersed the PEDOT chains, leading to a fragmented conductive network. The enhanced conductivity can be also confirmed by the leftward peak shifts in the Raman spectra (Fig. S9). Additionally, the processing parameters of the CW laser system also play a vital role in the conductivity of the film (Fig. 2i). With an increase in the applied laser energy density on the as-prepared MLLC-processed PEDOT:PSS films, the conductivity is gradually boosted due to the continuous enhancement of the phase separation. This can be confirmed by the Raman peak shift to the left (Fig. S10). The conductivity could be increased to 955 S/cm (average 881 S/cm) as the laser energy density reaches 2.93 J/cm^2^. However, a further increase in the laser intensity tends to degrade the film conductivity, caused by carbonization of the PEDOT:PSS (Fig. S11). Meanwhile, the effect of the laser-scanning speed on the film conductivity is similar to that of the laser power (Fig. S12). In short, optimization of the material composition and laser-processing parameters plays a crucial role in the film performance for the following bioelectronic applications.

### Mechanical and electrochemical characterizations

**Figure 3.**
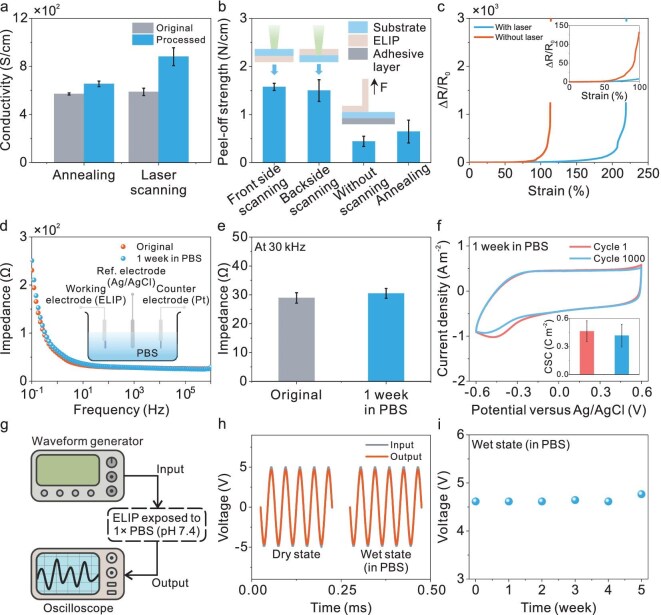
Mechanical and electrochemical properties of ELIP bioelectrodes. (a) Comparison of conductivity between annealing and laser-scanning processes (*n* = 5). (b) Interfacial adhesion forces of the PEDOT:PSS layer against the PET substrate under different processes (*n* = 5). The left two insets present the schematic of front-side and backside scanning. The right inset shows the method of standard 90-degree peeling test. (c) Relative resistance changes of ELIP hydrogels as a function of strain with and without laser scanning. Inset shows data for strains ranging from 0 to 100%. (d) EIS curves of the ELIP bioelectrodes before and after immersion in PBS solution for 1 week. Inset shows the schematic of the set-up with a platinum plate as the counter electrode and Ag/AgCl as the reference electrode. (e) Variation in the impedance magnitude of the ELIP bioelectrodes before and after immersion in PBS solution for 1 week at 30 kHz (*n* = 3). (f) CV curves of ELIP bioelectrodes measured in the 1st and 1000th cycles. Inset shows the corresponding changes in CSC (*n* = 3). (g) Schematic of the system to control input waveforms and monitor electrode output in PBS solution. (h) Output waveforms of bioelectrodes in dry and wet states, respectively. (i) Long-term changes in output amplitude of bioelectrodes immersed in PBS solution for 5 weeks.

Besides the conductivity, the mechanical and electrochemical properties were also evaluated. First, the thermal effect of the CW laser was investigated in comparison with traditional thermal annealing methods (Fig. 3a). It can be clearly observed that conductivity via the thermal annealing process undergoes a slight increase. In contrast, the conductivity of the MLLC films after laser treatment exhibits a much larger improvement from 588 to 881 S/cm. This implies the distinct merit of enhanced laser-induced phase separations that further optimize charge-transfer networks. To further evaluate the interfacial stability, a 90-degree peel-off test was performed to quantify the interfacial robustness against the substrate (Fig. 3b). The results reveal a remarkable increase in the peel-off strength of the MLLC films from 0.44 to 1.58 N/cm under laser scanning, suggesting that the laser treatment significantly strengthens the bonding between the ELIP hydrogels and the substrate. This surpassed the adhesion strength achieved by traditional thermal annealing, which only reached 0.65 N/cm. Interestingly, there was no notable difference in the peel-off strength between the front-side and backside laser scanning. This indicates that both front and back scanning can effectively enhance the adhesion force when the PEDOT:PSS layer is super thin (<1 μm). The change in the resistance as a function of the strain was also characterized (Fig. 3c). After laser treatment, the relative resistance of the ELIP hydrogels underwent an obvious change at a strain of ∼220%, demonstrating a much higher strain threshold compared with that of the untreated films. This impressive strain tolerance shows the robustness of the electrical properties under mechanical deformation, ensuring that the device can maintain functionality in stretchable bioelectronic applications.

Electrochemical capabilities in physiological environments are also essential to ensure long-term functionality in implanted measurement. To evaluate these properties, cyclic voltammetry (CV) and electrochemical impedance spectroscopy (EIS) were carried out by using a three-electrode model, with the proposed electrode as the working electrode, a platinum plate as the counter electrode and Ag/AgCl as the reference electrode. This configuration allows evaluation of the stability and electrochemical behaviors of bioelectrodes under conditions that mimic biological systems. First, comparisons of the impedance and phase before and after soaking in phosphate buffer solution (PBS) for 1 week are shown in Fig. 3d and Fig. S13. It can be seen that the increases are almost negligible after soaking in PBS solution for 1 week. For example, the impedance of the ELIP hydrogels at 30 kHz slightly increases from 28.9 to 30.5 Ω after soaking (Fig. 3e). In addition, it is noted that the modified ELIP exhibits superior long-term stability, presenting almost no change in electrical performance after being stored for >4 months (Fig. S14). Meanwhile, the ELIP hydrogel reached a steady state within 1 h in an acid environment (pH = 4) and could maintain its properties for 24 h (Fig. S15). Such stability in impedance is critical for bioelectronic applications in which consistent electrical performance is crucial for reliable and long-term signal transmission and stimulation.

Additionally, to simulate the demands of repeated electrical stimulation, the ELIP hydrogels underwent 1000 charge–discharge cycles to assess their durability in electron injection and ejection processes (Fig. 3f). The charge storage capacity (CSC) was calculated from the data (inset of Fig. 3f), which shows a slight decrease from 0.46 to 0.42 C/m^2^ after 1000 repeated cycles. This minor decrease suggests that the film maintains a robust interfacial structure, probably due to a stable interlocking mechanism between the active layer and its substrate [35]. This interlocking prevents structural disintegration and delamination, which may take place due to the repeated volumetric changes that accompany charge and discharge cycles. Overall, these findings indicate that the proposed ELIP hydrogels are endowed with superior electrochemical stability, rendering them as promising conductive nanomaterials for long-term bioelectronic applications.

Moreover, to evaluate the stimulation capability of ELIP hydrogels in a physiological environment, they were exposed to PBS (pH 7.4) solution and subjected to alternating current (AC) electrical stimulation (Fig. 3g). This model was applied to mimic conditions that are similar to those in the body, allowing an *in vivo* assessment of performance in practical applications. By comparing the input and output waveforms, it is clearly observed that the stable and consistent output can be transmitted via the proposed bioelectrode at both dry and wet states (Fig. 3h). For a long-term stability test, the bioelectrode was immersed in PBS solution for 5 weeks and received electrical stimulation for 12 h per day. Figure 3i illustrates that the ELIP hydrogels still maintained a stable output voltage throughout this extended period, highlighting their superior durability and conductivity even under continuous stimulation. This sustainable performance under physiological conditions highlights the merits of the proposed bioelectrode as a robust and reliable material for electrical stimulation applications.

### Demonstrations of nerve-conduction block and biocompatibility assessment

**Figure 4.**
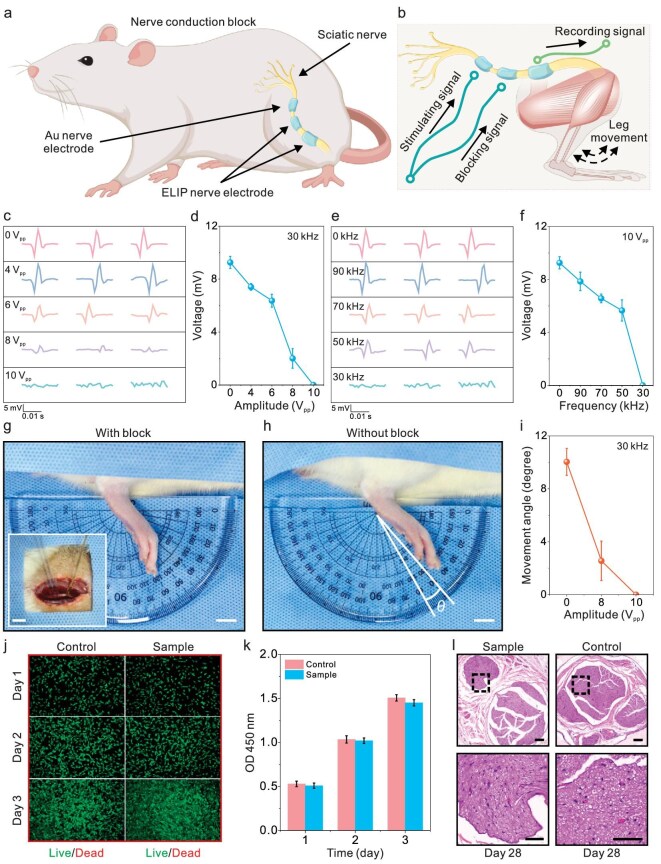
Demonstration of nerve-conduction block and biocompatibility test. (a) Schematic of the cuff electrode configuration to evaluate nerve-conduction block. (b) Rat model with three cuff electrodes to stimulate, block and record nerve signals by observing leg movement. (c) Captured action potentials and (d) the effect of blocking signal amplitude (0–10 V_pp_) on nerve action potentials (*n* = 5). (e) Captured action potentials and (f) the effect of blocking signal frequency (0–90 kHz) on nerve action potentials (*n* = 5). (g and h) Photos of ankle joint movement angle with and without nerve-conduction block. Scale bars, 10 and 5 mm (inset). (i) Changes in ankle joint movement angle at different blocking parameters. (j) Cell morphology and viability comparison between the proposed bioelectrodes and control groups using fluorescence microscopy images and (k) cell survival rate based on absorbance at 450 nm (*n* = 3). (l) Histological analysis of nerve after 28 days of implantation of the proposed cuff electrodes (top) and corresponding magnified images (below). Scale bars, 200 μm (top) and 50 μm (below).

To showcase the potential applications of the ELIP hydrogels for implantable scenarios, an *in*  *vivo* demonstration of nerve-conduction block was performed to exhibit the capacity to stimulate and record nerve signals (Fig. 4a). Nerve-conduction block is a neuromodulation technique that temporarily interrupts the transmission of action potentials, rendering it an effective approach for pain relief. Specifically, nerves can maintain a dynamic steady-state depolarization by applying kilohertz-frequency AC [41]. Such a state enables the conduction of action potentials that carry pain signals to be blocked. Compared with drug-based treatments, nerve-conduction block reduces pain with minimal side effects to health. This is considered to be an effective approach for relieving acute pain.

To be specific, a rat model that had three cuff electrodes to stimulate, block and record nerve signals was employed to investigate the effect of the proposed bioelectrodes in nerve-conduction block for pain management (Fig. 4b). Among them, a gold electrode served as the stimulator to efficiently trigger action potentials. The ELIP bioelectrodes acted as the blocking and recording electrodes to study the principle of nerve-conduction block. First, the effect of amplitude (0–10 V_pp_) and frequency (0–90 kHz) of the blocking signals was investigated by recording the nerve action potentials. By increasing the amplitude and decreasing the frequency of the blocking signal, a significant reduction could be observed in the nerve action potentials (Fig. 4c–f). Higher blocking signal amplitudes led to a remarkable decrease in the recorded nerve action potentials, while the lower frequency also contributed to the attenuation. At an optimized blocking frequency of 30 kHz or an amplitude of 10 V_pp_, the nerve action potentials became almost undetectable, presenting effective signal suppression.

Building upon these *in situ* electric measurements, movement of an ankle joint was evaluated as an indicator of nerve function. According to the real-time results, the ankle movement of the rat decreased from ∼10 degrees to nearly zero, indicating almost complete inhibition of movement under the optimized conditions (Fig. 4g–i and Supplementary Video S1). This physical observation further implies the potential of the proposed bioelectrodes in blocking the nerve signals that are responsible for pain transmission. These results suggest that PEDOT-based nerve-conduction blocks offer a promising, non-pharmacological alternative for pain treatments, thus reducing the reliance on drug-based interventions for pain relief.

Considering the scenarios of implantable applications, tests were also conducted to assess the biocompatibility and safety of the proposed bioelectrodes. First, the cell toxicity of the bioelectrodes was evaluated by using *in vitro* experiments (Fig. 4j). All the samples were exposed to ultraviolet light for 30 min before testing. This showed that the cell density and morphology of the sample group were similar to those of the control group with the live/dead cell assay during 3 days. Furthermore, the cell survival rate of each group was calculated based on the absorbance value at 450 nm (Fig. 4k and Fig. S16). As can be seen, the ELIP bioelectrodes exhibited no discernible impact on the cellular viability, demonstrating their low cytotoxicity. In addition, the *in vivo* responses to nerve damage and tissue inflammation were evaluated by implanting cuff bioelectrodes on the sciatic nerve for 28 days. The control group underwent the same surgery while no materials were implanted. Throughout the entire process of electrode implantation and explantation, the rats exhibited normal behavior without any signs of physiological abnormalities. Histological analysis revealed normal nerve and muscle tissue morphology without signs of inflammation or nerve damage, verifying their desired biocompatibility (Fig. 4l and Figs S17 and S18). In short, these findings indicate that the proposed bioelectrodes exhibit almost no cytotoxicity and inflammatory response *in vivo*, supporting their biomedical applications as safe and effective conductive nanomaterials for bioelectronic interfaces.

## DISCUSSION

This work presents an enhanced laser-induced PEDOT:PSS-based hydrogel that overcomes the limitations of weak photothermal conversion. By employing MLLC processing, the pretreated PEDOT:PSS exhibits significantly improved absorbance due to the increase in the conjugation length in the PEDOT domain as revealed by molecular dynamics calculations. This contributes to intensive photothermal conversion in the visible-light region, achieving the desired conductivity. Moreover, the proposed bioelectrodes showcase superior electrochemical stability and interfacial mechanical robustness, maintaining consistent function in physiological environments. The femtosecond laser-based subtractive process enables high-precision patterning down to 3 μm. Demonstrations of nerve-conduction block highlight their potential for neurological applications in neural interfaces and pain management, with desired conductivity and biocompatibility.

## METHODS

Detailed materials and methods are available in the Supplementary data.

## ETHICAL STATEMENTS

This study was performed in accordance with the recommendations in the Guide for the Care and Use of Laboratory Animals and relevant Chinese laws and regulations. All procedures were approved by the Laboratory Animal Welfare and Ethics Review Committee of Zhejiang University (ZJU20240573).

## Supplementary Material

nwaf136_Supplemental_File
