# Correction to: Enhanced laser-induced PEDOT-based hydrogels for highly conductive bioelectronics

**DOI:** 10.1093/nsr/nwag080

**Published:** 2026-03-11

**Authors:** Hao Zhou, Ziguan Jin, Yuhong Xu, Yuyao Lu, Zhuoheng Xia, Fan Yang, Qianglong Wu, Yang Gao, Jun Yin, Jianhua Zhang, Chujun Ni, Bin Zhang, Yong He, Huayong Yang, Kaichen Xu

**Affiliations:** State Key Laboratory of Fluid Power & Mechatronic Systems, School of Mechanical Engineering, Zhejiang University, Hangzhou 310023, China; State Key Laboratory of Fluid Power & Mechatronic Systems, School of Mechanical Engineering, Zhejiang University, Hangzhou 310023, China; State Key Laboratory of Fluid Power & Mechatronic Systems, School of Mechanical Engineering, Zhejiang University, Hangzhou 310023, China; State Key Laboratory of Fluid Power & Mechatronic Systems, School of Mechanical Engineering, Zhejiang University, Hangzhou 310023, China; Center for Plastic & Reconstructive Surgery, Department of Stomatology, Zhejiang Provincial People’s Hospital, Affiliated People’s Hospital, Hangzhou Medical College, Hangzhou 310023, China; Center for Plastic & Reconstructive Surgery, Department of Stomatology, Zhejiang Provincial People’s Hospital, Affiliated People’s Hospital, Hangzhou Medical College, Hangzhou 310023, China; Center for X-Mechanics, Department of Engineering Mechanics, Zhejiang University, Hangzhou 310027, China; Center for X-Mechanics, Department of Engineering Mechanics, Zhejiang University, Hangzhou 310027, China; State Key Laboratory of Fluid Power & Mechatronic Systems, School of Mechanical Engineering, Zhejiang University, Hangzhou 310023, China; State Key Laboratory of Fluid Power & Mechatronic Systems, School of Mechanical Engineering, Zhejiang University, Hangzhou 310023, China; Eye Center, Affiliated Second Hospital, School of Medicine, Zhejiang University, Hangzhou, 310009, China; State Key Laboratory of Fluid Power & Mechatronic Systems, School of Mechanical Engineering, Zhejiang University, Hangzhou 310023, China; State Key Laboratory of Fluid Power & Mechatronic Systems, School of Mechanical Engineering, Zhejiang University, Hangzhou 310023, China; State Key Laboratory of Fluid Power & Mechatronic Systems, School of Mechanical Engineering, Zhejiang University, Hangzhou 310023, China; State Key Laboratory of Fluid Power & Mechatronic Systems, School of Mechanical Engineering, Zhejiang University, Hangzhou 310023, China

In the Fig. 1 of ‘Enhanced laser-induced PEDOT-based hydrogels for highly conductive bioelectronics’ (*National Science Review*, Volume 12, Issue 6, 2025, nwaf136, https://doi.org/10.1093/nsr/nwaf136), two errors were identified. In Fig. 1b, the X-axis label should be corrected from ‘Strain (%)’ to ‘Wavelength (nm)’. In Fig. 1e, the scale bar should be corrected from ‘10 μm’ to ‘10 mm’.

**Figure 1.**
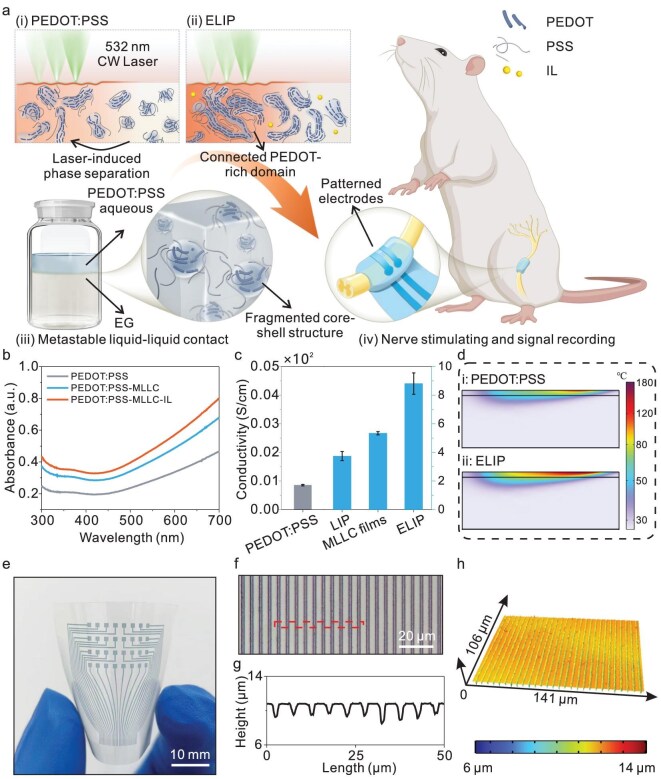
Design and fabrication of highly conductive hydrogels. (a) Schematic of design principles of bioelectrodes via MLLC coupled with laser-induced phase separation for implantable nerve stimulation and signal recording. (b) Ultraviolet-visible absorption spectra of pure PEDOT:PSS, MLLC ink and MLLC ink optimized with ionic liquid (IL). (c) Electrical conductivity of PEDOT:PSS and MLLC films with and without laser processing (*n* = 5). (d) Calculated thermal distribution inside the PEDOT:PSS and MLLC films. (e) Photo of enhanced laser-induced PEDOT:PSS electrodes array patterned on a PET substrate. (f) Optical image, (g) corresponding height profile and (h) 3D topography of patterned ELIP bioelectrodes array with a width of ∼3 μm on the PET substrate.

The corrected Fig. 1 is presented below. We sincerely apologize for any inconvenience caused. This correction does not affect the conclusions of the article.

