# Supplementary material for: Enhanced laser-induced PEDOT-based hydrogels for highly conductive bioelectronics
Source: Natl Sci Rev. 2025 Apr 4;12(6):nwaf136. doi: 10.1093/nsr/nwaf136 (PMC12086669; doi:10.1093/nsr/nwaf136)
Supplement: nwaf136_Supplemental_File [file nwaf136_supplemental_file.pdf]

Supplementary Information

Enhanced laser-induced PEDOT-based hydrogels for highly conductive bioelectronics

Hao Zhou[1], Ziguan Jin[1], Yuhong Xu[1], Yuyao Lu[1], Zhuoheng Xia[2], Fan Yang[2], Qianglong Wu[3], Yang Gao[3], Jun Yin[1], Jianhua Zhang[1], Chujun Ni[4], Bin Zhang[1], Yong He[1], Huayong Yang[1] and Kaichen Xu[1,*]

[1]State Key Laboratory of Fluid Power & Mechatronic Systems, School of Mechanical Engineering, Zhejiang University, Hangzhou 310023, China;

[2]Center for Plastic & Reconstructive Surgery, Department of Stomatology, Zhejiang Provincial People's Hospital, Affiliated People's Hospital, Hangzhou Medical College, Hangzhou 310023, China;

[3]Center for X-Mechanics, Department of Engineering Mechanics, Zhejiang University, Hangzhou 310027, China;

[4]Eye Center, Affiliated Second Hospital, School of Medicine, Zhejiang University, Hangzhou, 310009, China

***Corresponding author**. E-mail: xukc@zju.edu.cn

Supplementary Note 1. Methods

Preparation of the optimized PEDOT:PSS-based ink

PEDOT:PSS aqueous solution (Clevios PH1000) was purchased from Heraeus and filtered through 0.45-μm-polyvinylidene-fluoride-filters after stirring. The PEDOT:PSS solution was then gradually dropped onto the surface of ethylene glycol (EG, Aladdin) solution. After 4 hours at room temperature, the upper MLLC-processed PEDOT:PSS was collected. Then, 50 wt% ionic liquid (4-(3-butyl-1-imidazolio)-1-butanesulfonic acid triflate, Dibai) was added and stirred for 3 hours at room temperature to obtain the ink for the subsequent use.

Enhanced laser-induced PEDOT-based films and subtractive patterning

First, the substrates like glass, PET, and TPU were exposed to oxygen plasma for 10 min after cleaning. The as-prepared ink was then spin coated onto the substrates and dried at 130 °C for 10 min. Subsequently, the resultant films were treated by a CW laser (wavelength of 532 nm) processing system, with laser energy densities ranging from 0 to 5.85 J cm^{-2} and scanning speeds between 0 and 400 mm s^{-1}. The pure PEDOT:PSS films were processed by the optimized laser parameters of 2.93 J cm^{-2} and 100 mm s^{-1}, respectively. Finally, a femtosecond laser system was employed to prepare patterned bioelectrodes, with the optimized parameters of a pulse fluence of 7.0 J cm^{-2} and a scanning speed of 1 mm s^{-1}.

Finite element simulations

Finite element analyses for simulating the thermal field of laser irradiation on the original PEDOT:PSS and MLLC films were carried out by using a commercial package COMSOL 6.2. The solid heat transfer module in COMSOL was used to simulate the temperature distribution within the materials by setting related laser parameters. It is challenging to precisely measure or calculate the temperature distribution within the films in experiments or simulations. Therefore, the emphasis was placed on the temperature differences between the PEDOT:PSS and ELIP hydrogels under the same laser parameters (laser radius, 5 μm; laser power, 8 mW; laser speed, 300 mm s^{-1}). The thermal conductivity of MLLC films was conducted by a thermal conductivity meter (Hot Disk TPS2500S, Hotdisk, Sweden). Specific heat capacity was evaluated using the DSC sapphire method (TA DSC25, TA Instruments, USA).

Material characterizations

An optical microscope (MCK-6RC, Caikon, China) was applied to capture images of the patterned ELIP hydrogels. 3D topographies and thickness of ELIP hydrogels were measured by a laser confocal microscope (OLS5000, Olympus, Japan). Surface topography was examined by AFM (Asylum Research MFP-3D-Bio, Oxford instrument, UK). Optical properties were evaluated using an ultraviolet-visible (UV-Vis) spectrophotometer (UV-3600, Shimadzu, Japan). Chemical structure analysis was conducted by XPS analysis (K-Alpha, Thermo Scientific, USA) and Raman spectroscopy (LabRAM Soleil, Horiba, France).

Electrical and mechanical characterizations

The sheet resistance (R) of different types of films on the glass substrate was measured by a multimeter. By taking into account the thickness (d) of films, the electrical conductivity (σ) could be calculated by the equation: $\sigma = 1/R \cdot d$. The hydrated ELIP films were prepared by soaking in DI water for one hour. The change of electrical resistance of the ELIP hydrogels on the TPU substrate under various strains was recorded by a benchtop multimeter (34470A, Keysight, USA) and a tensile testing machine (FSA-0.5K2-500 N, IMADA). The change of electrical resistance of the ELIP hydrogels on the PET substrate in an acid environment (pH=4) was recorded by the benchtop multimeter.

Adhesion characterization

First, the MLLC-processed PEDOT:PSS ink was spin coated onto the PET substrate. Then, epoxy adhesive (DP420, 3M) was covered onto the interface between the ELIP hydrogels and glass substrate. After that, the adhesion on the interface between the ELIP hydrogels and PET substrate was measured through a 90° peel-off test by using a tensile testing machine.

Electrochemical and stability characterization

The EIS and CV tests were performed by an electrochemical workstation (CHI760E, CH Instruments Ins.) with a platinum plate as the counter electrode and Ag/AgCl as the reference electrode. Before testing, half of the samples were immersed in PBS solution for one week at room temperature. The EIS was tested from 0.1 Hz to 1 MHz with an AC perturbation amplitude of 5 mV. With a scan rate of 0.02 V s^{-1}, the CV tests were recorded between -0.6 and 0.6 V. Additionally, CSC values were calculated from each CV data. For the stability test during AC electrical stimulation, the proposed conductive film (1 × 3 cm)

was soaked in PBS solution at room temperature for 5 weeks. Then, 12 hours of AC stimulation per day (25 kHz, 5 V) by using a signal generator was applied onto the film. The output was recorded using an oscilloscope.

MD models

Molecular dynamics simulations were carried out using Gromacs 2018.8 program with general Amber force fields (GAFF). All molecules were optimized by Gaussian 16 program using M06-2X hybrid functional theory with 6-31G* basis set. Multiwfn was used to construct RESP charges. The PSS chain of 6 repeating units and the PEDOT chain consisting of 36 repeating units were prepared, respectively. Then two separate cubic boxes were constructed by using packmol software with a side length of 10 nm. The first box contained 1 PEDOT molecule, 6 PSS molecules, and 20000 water molecules. The second box comprised 1 PEDOT molecule, 6 PSS molecules, 7500 ethylene glycol molecules and 1500 water molecules. The cut-off for neighbor list of Verlet method and for short-range interactions was set to 1.2 nm in all calculations with periodic boundary conditions in three directions. After the energy minimization, the systems were equilibrated in NPT ensemble. In this simulation, the system was simulated for 200 ps using Berendsen thermostat at 298.15K with a coupling constant of 0.1 ps. Then, all systems finally ran in NVT ensemble for 10 ns at 298.15K. The time step of each simulation was set to 2 fs.

In vitro cytotoxicity characterization assay

The medium consisted of 89% Dulbecco's modified Eagle's medium (DMEM, BL304A, Biosharp), 10% fetal bovine serum (FBS, BS105, OPCELL), and 1% streptomycin/penicillin (P/S, C0222, Biyuntian). HT22 cell suspension was cultured in the medium adaptively for two iterations. Based on the result of cell count, the cell density was adjusted to 3×10^4 cells mL^{-1}. Then, the cells were seeded at 100 μL per well in three 96-well plates. Every plate was split up into two groups, each of which contained three duplicates. Additionally, a blank well containing only complete medium served as a control, while the remaining wells were supplemented with PBS and cultured for 24 hours.

For the experimental groups, the enhanced laser-induced samples were exposed to UV light for 30 minutes. Then, the samples were soaked in the medium at a density of 6 cm^2 mL^{-1} at 37 °C for 24 hours. After soaking, the samples were filtered through a 0.22 μm filter for testing. In addition, the control group

received the same treatment in complete medium.

The original medium was removed from the wells, and the wells were subsequently filled with the fresh complete culture medium as prepared in the immersion step. All the cells were incubated continuously for 1, 2, and 3 days. At each of three time points, the culture medium was removed and 100 μL of CCK-8 solution (90% DMEM + 10% CCK-8 reagent) was added to each well. Empty wells without cells acted as the blank control. After incubating for a specific period, the absorbance at 450 nm was measured by using a microplate reader. The data were recorded as OD_{Sample}, $OD_{Control}$, and OD_{Blank}, respectively. Based on the absorbance data, the cell viability of each group could be calculated by the formula,

$$\text{Cell Viability (\%)} = 100\% \times \frac{(OD_{Sample} - OD_{Blank})}{(OD_{Control} - OD_{Blank})}$$

In vivo histological biocompatibility test

Firstly, a 1 × 18 mm bioelectrode was prepared on a 3 × 20 mm TPU substrate. Due to the self-adhesive property of TPU, the bioelectrode was placed as a cuffed electrode on the sciatic nerve of the healthy rat following 30 minutes of UV irradiation. Additionally, the rats in the blank group underwent similar surgery, but no electrodes were implanted.

12 adult male Lewis rats were euthanized through CO_2 gas at 1, 2, and 4 weeks, respectively. Then, the sciatic nerve with samples and surrounding gastrocnemius muscle were extracted. After that, such samples were fixed in 4% paraformaldehyde overnight and then transferred to PBS for histological analyses. For immunofluorescence staining, all the samples were analyzed by H&E staining after paraffin embedding and sectioning.

Electrical stimulation and neural signal recording

The neuroelectric stimulation signals (amplitude of 10 mV, frequency of 200 Hz) were generated by a signal generator (Agilent, 33522A). In addition, the stimulation electrodes were made of Au electrodes. For blocking electrodes, rectangular ELIP bioelectrodes (width, 1 mm) were patterned onto the TPU substrate and exposed under UV irradiation for 30 min. Under sterile conditions, the sciatic nerve of rat with enough length was exposed for implanting cuffed electrodes. Blocking signals were also generated by the signal generator with a peak-to-peak voltage of 0-10 V_{pp} and a frequency of 0-90 kHz.

The recording electrodes were composed of the ELIP bioelectrodes. The signals were recorded by a homemade data acquisition module, which mainly consisted of an A/D converter, a microcontroller with a built-in low-power Bluetooth module. Such system was capable of collecting physiological signals with a sampling rate of 1000 Hz and wirelessly transmitting them to a receiver. The signals could be displayed in real time on a user interface.

Statistical Analysis

All the statistical results were analyzed by Origin software and depicted as means ± standard deviations ($n \geq 3$). Cytotoxicity, histological and immunohistochemical analyses were quantitatively performed with three samples per each group.

Supplementary Note 2. Theoretical study of spatial heat transfer under laser interaction

Assuming the laser intensity on the material surface following a Gaussian distribution, it can be expressed as

$$I(x,y) = I_0 \exp\left(-\left(\frac{x^2}{2\sigma_x^2} + \frac{y^2}{2\sigma_y^2}\right)\right) \quad for\ \sqrt{x^2+y^2} \leq \omega \tag{1}$$

wherein, $I(x,y)$ is the light intensity at position (x,y), I_0 is the maximum intensity at the center of the beam, ω is the beam radius, σ_x and σ_y are the standard deviations of the Gaussian distribution in the x and y directions, respectively. For a circularly symmetric Gaussian beam, there is $\sigma_0 = \sigma_x = \sigma_y$. Equation (1) can be simplified as

$$I(x,y) = I_0 \exp\left(-\frac{x^2+y^2}{2\sigma_0^2}\right) \tag{2}$$

Rayleigh length z_R represents the ability of beam to remain collimated within a certain depth range and is given by the formula as

$$z_R = \frac{\pi\omega_0^2}{\lambda} \tag{3}$$

where λ and ω_0 are the wavelength and beam waist radius of the laser, respectively. The beam radius $\omega(z)$ at depth z can be expressed as

$$\omega(z) = \omega_0\sqrt{1+\left(\frac{z}{z_R}\right)^2} \tag{4}$$

Extending the Gaussian intensity distribution to three-dimensional space, the intensity distribution of the Gaussian beam at depth z can be expressed as

$$I(x,y,z) = I_0 \left(\frac{\omega_0}{\omega(z)}\right)^2 \exp\left(-\frac{x^2+y^2}{\omega(z)^2}\right) \tag{5}$$

wherein, I_0 is the maximum intensity of the beam at the focal plane. At the focal plane (z = 0), the maximum intensity I_0 of the Gaussian beam can be calculated by using the total power P and the beam waist radius ω_0 as

$$P = \iint I(x,y)\, dx\, dy = \iint I_0 \exp\left(-\frac{x^2+y^2}{\omega_0^2}\right) dx\, dy \tag{6}$$

The maximum intensity can be calculated as

$$I_0 = \frac{2P}{\pi\omega_0^2} \tag{7}$$

By combining all the formulas above, the intensity distribution at any depth z can be expressed as

$$I(x,y,z) = \frac{2P}{\pi\omega_0^2}\left(\frac{\omega_0}{\omega(z)}\right)^2 \exp\left(-\frac{x^2+y^2}{\omega_0^2}\right) \tag{8}$$

This formula describes the intensity distribution of a CW laser with power P and wavelength λ at different depths and positions inside the material. Absorbance A represents the material's ability to absorb the laser. Supposing that the absorbed laser intensity I_{abs} is given by

$$I_{abs}(x,y,z) = A \cdot I(x,y,z) \tag{9}$$

The spatial heat transfer under laser interaction can be described as

$$Q(x,y,z,t) = A \cdot I_0 \left(\frac{\omega_0}{\omega(z)}\right)^2 exp\left(-\frac{\left(x-\mu_x(t)\right)^2+\left(y-\mu_y(t)\right)^2}{\omega(z)^2}\right) \cdot t \tag{10}$$

where Q is the heat within the film at position (x,y,z) at time t, I_0 is the maximum intensity at the center of the beam, ω is the radius of the light spot, $(\mu_x(t), \mu_x(t))$ is center coordinates at time t.

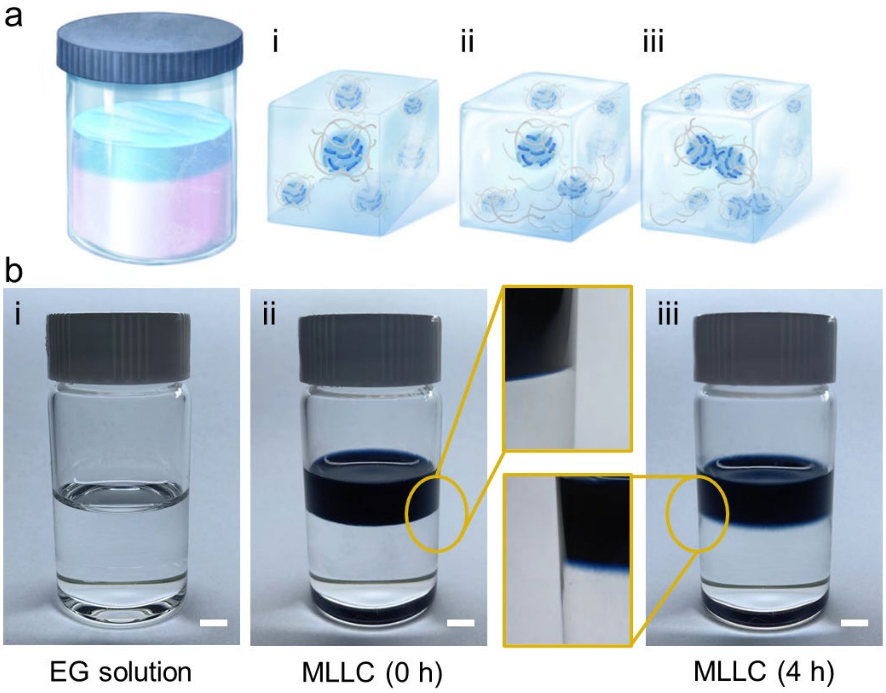

Fig. S1. Schematics (a) and photos (b) of the MLLC processing. Scale bar, 5 mm.

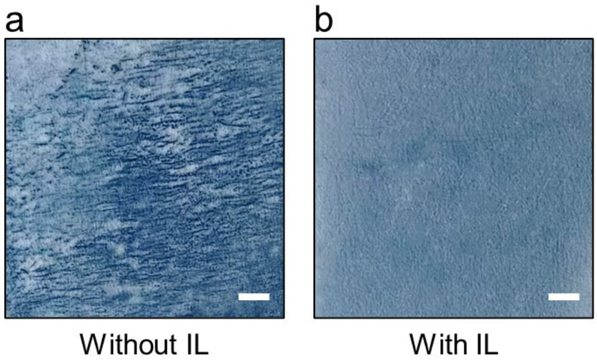

Fig. S2. Photos of MLLC films with and without IL. Scale bar, 2 mm.

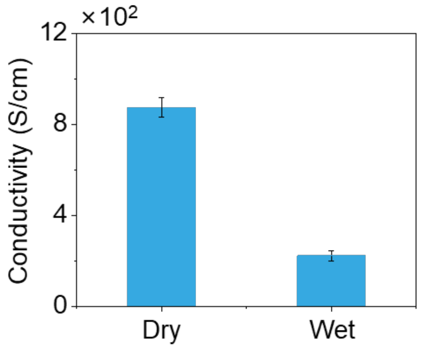

Fig. S3. Electrical conductivity of ELIP hydrogels in dry and wet state.

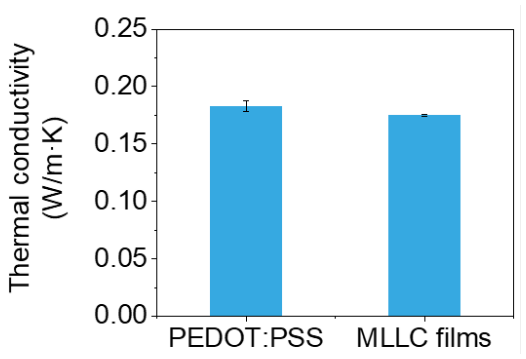

Fig. S4. Thermal conductivity of PEDOT:PSS and MLLC films.

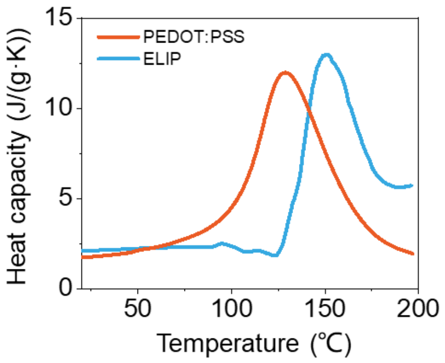

Fig. S5. Heat capacity of PEDOT:PSS and MLLC films.

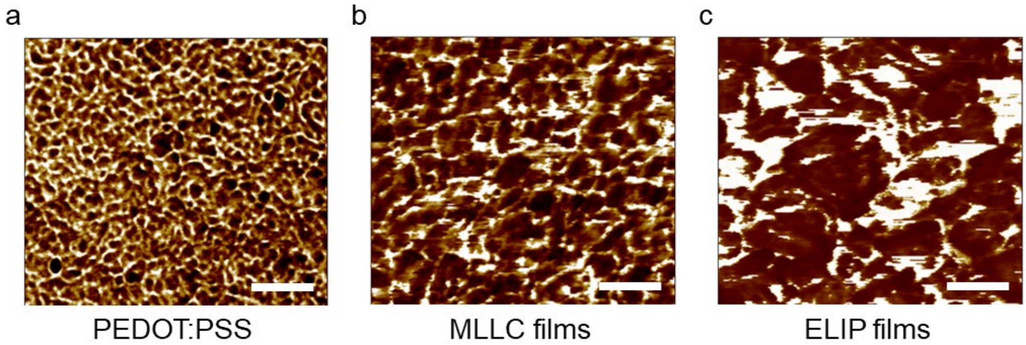

Fig. S6. AFM images of PEDOT:PSS films at each fabrication stage. Scale bar, 200 nm.

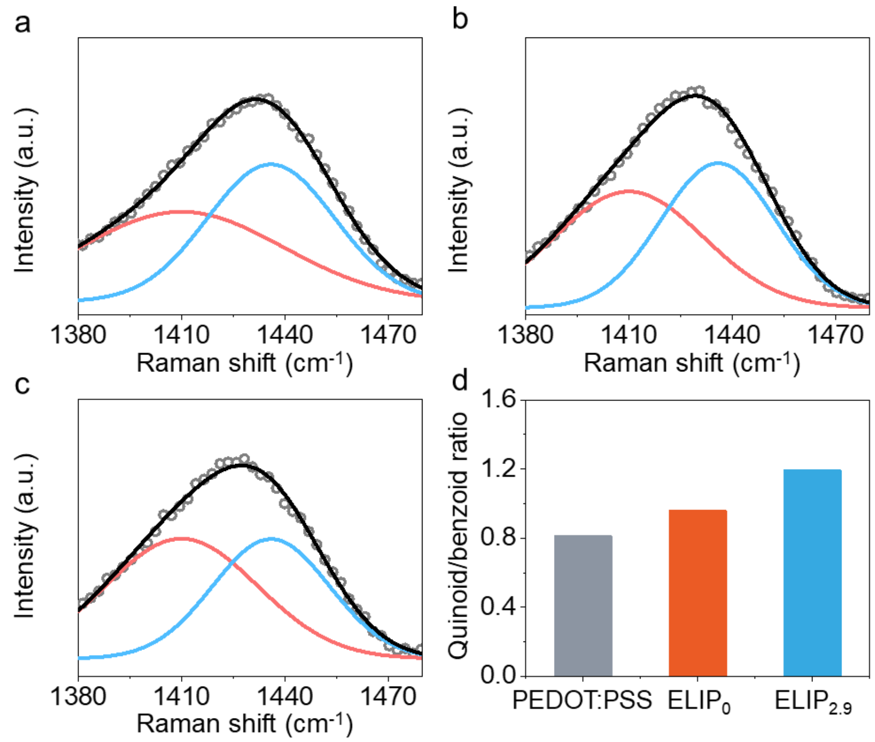

Fig. S7. Fitted Raman spectroscopy results of (a) PEDOT:PSS, (b) MLLC films, (c) ELIP hydrogels, and (d) corresponding comparison in quinoid/benzoid ratio.

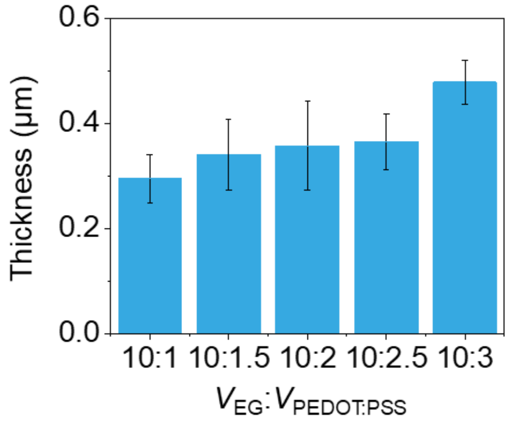

Fig. S8. Thickness of MLLC samples according to the different volume ratio of EG to PEDOT:PSS**.**

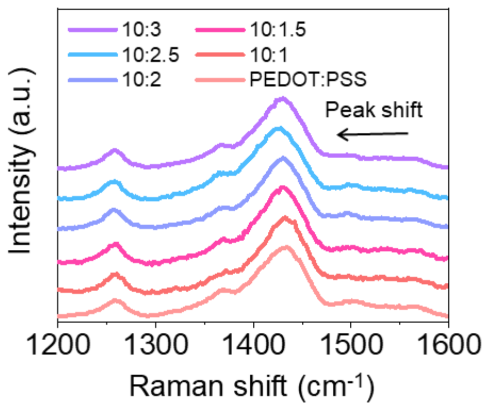

Fig. S9. Raman spectroscopy results according to the different volume ratios of EG to PEDOT:PSS for the ELIP hydrogels**.**

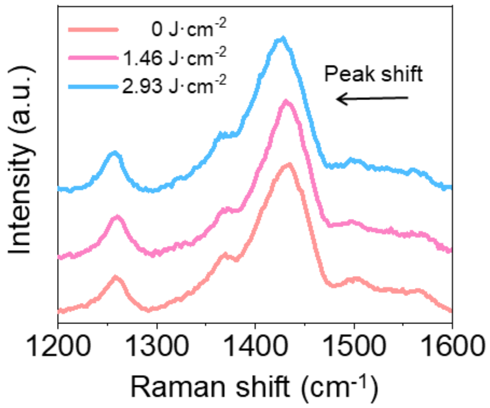

Fig. S10. Raman spectroscopy results according to the different laser energy densities for the ELIP hydrogels**.**

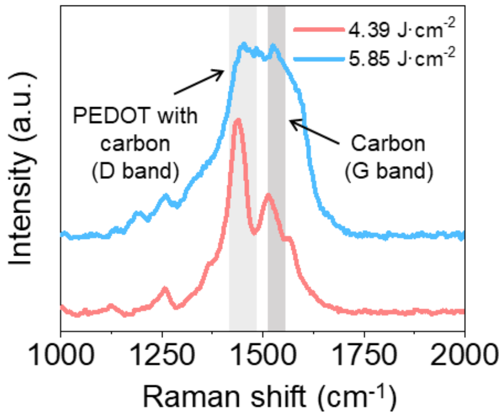

Fig. S11. Raman spectroscopy results of carbonized ELIP samples.

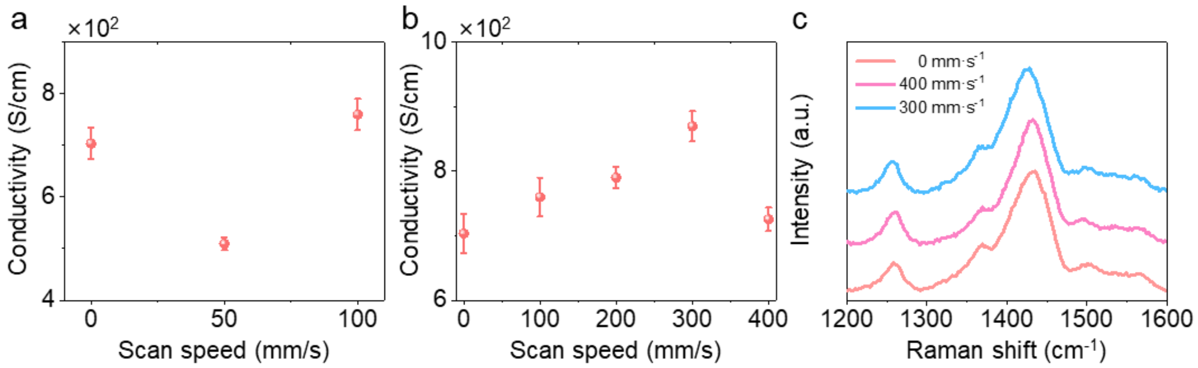

Fig. S12. (a, b) Conductivity of ELIP film at different scan speeds, and (c) corresponding Raman spectroscopy results.

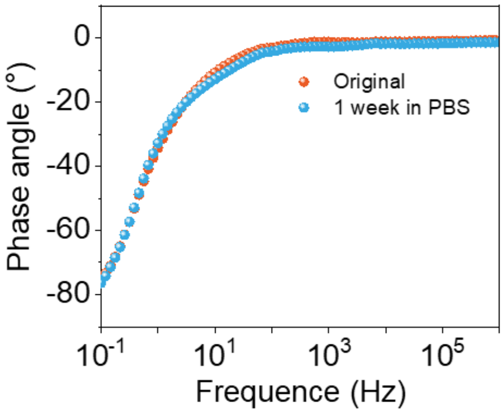

Fig. S13. Phase curves of ELIP hydrogels before and after soaking in PBS solution for one week.

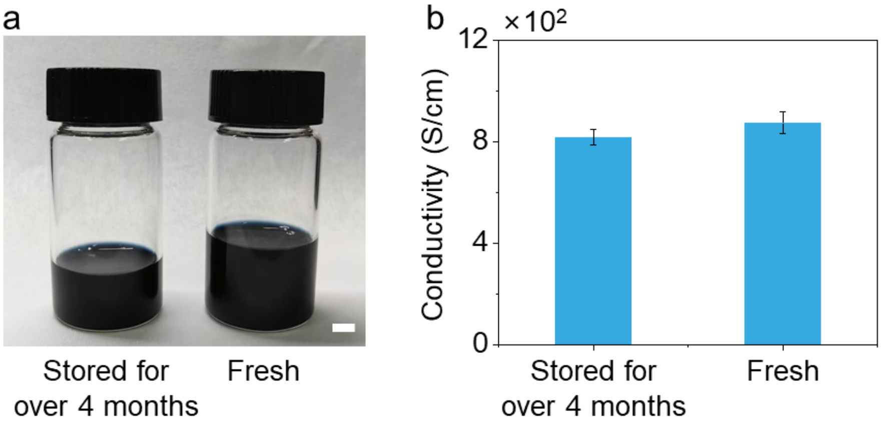

Fig. S14. (a) Photos and (b) electrical conductivity of MLLC inks after storage for over 4 months and freshly prepared samples. Scale bar, 5 mm.

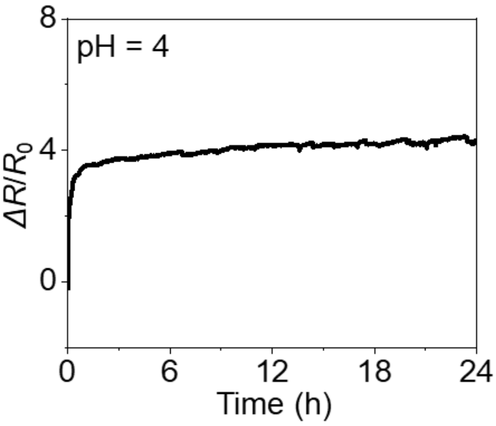

Fig. S15. Relative resistance changes of ELIP hydrogels after immersing in an acid environment (pH=4) for 24 hours.

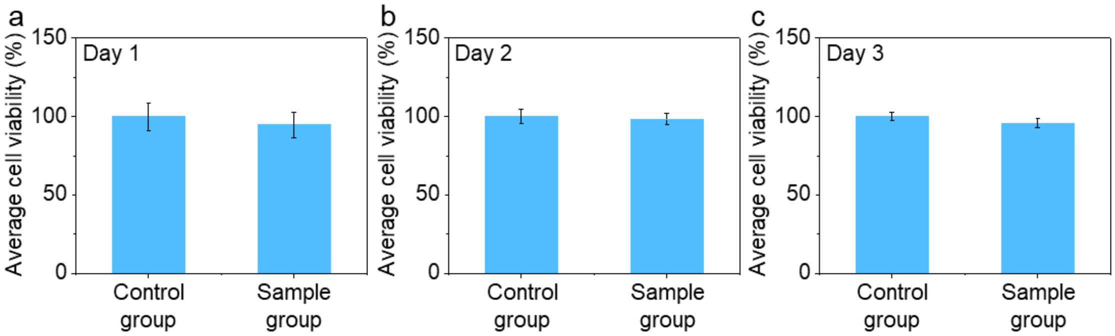

Fig. S16. Comparison of average cell viability of control and sample groups for each day.

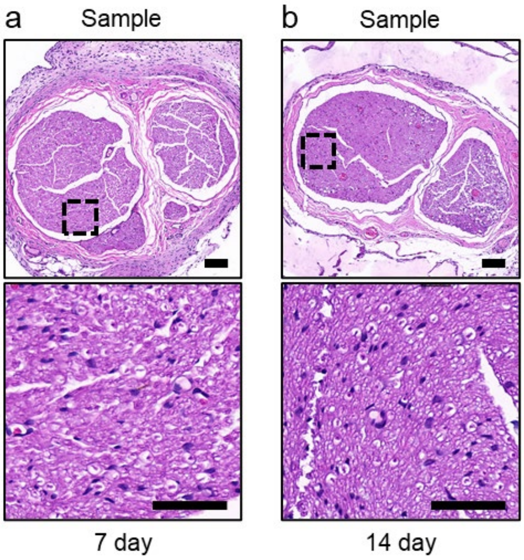

Fig. S17. Histological analysis of nerve after 7 days (a) and 14 days (b) implantation of ELIP cuff electrodes (top) and corresponding zoomed images (below). Scale bar, 200 μm (top) and 50 μm (below).

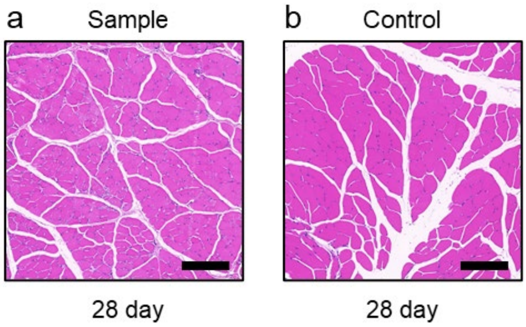

Fig. S18. Histological analysis of gastrocnemius muscle in the sample (a) and control (b) group after 28 days implantation. Scale bar, 200 μm.
